# How can midwives in Germany be supported in advising on early childhood allergy prevention in a health literacy-responsive way? Protocol for a mixed-methods study to co-design and evaluate an educational intervention following the Medical Research Council framework

**DOI:** 10.1136/bmjopen-2024-098402

**Published:** 2025-12-11

**Authors:** Julia von Sommoggy, Julia Ruth Steinmann, Jonas Lander, Eva Maria Bitzer, Maja Pawellek, Susanne Brandstetter, Christian Apfelbacher, Barbara Dorothea Fillenberg

**Affiliations:** 1 Institute of Social Medicine and Health Systems Research, Faculty of Medicine, Otto von Guericke University, Magdeburg, Germany; 2 Midwifery Science, Johannes Gutenberg University, Mainz, Germany; 3 Institute for Epidemiology, Social Medicine and Health Systems Research, Hannover Medical School, Hannover, Germany; 4 Public Health and Health Education, University of Education, Freiburg, Germany; 5 Hospital St. Hedwig of the Order of St. John, University Children’s Hospital Regensburg (KUNO), Regensburg, Germany

**Keywords:** Health Literacy, PREVENTIVE MEDICINE, Midwifery

## Abstract

**Abstract:**

**Introduction:**

Health literacy (HL) is essential for making informed health-related decisions, for example enabling parents to reduce their child’s allergy risk. Health literacy does not, however, rely solely on an individual’s capacities, but is strongly influenced by external factors. Midwives provide important health advice to families, particularly since their relationship is close during a time of significant transition. This offers them a unique opportunity to positively influence the HL of parents, which in turn may support the health and well-being of the whole family. The aim of this study is to develop and evaluate an intervention that can support midwives in providing allergy prevention advice in a way that is in line with the concept of HL.

**Methods and analysis:**

In accordance with the recommendations of the Medical Research Council framework in the first phase of this study, we will survey midwives (target sample size=379) in Germany regarding their practices, the potential barriers they face and enabling factors in providing advice on early childhood allergy prevention in an HL-responsive way. The data will be subjected to descriptive statistical analysis. Two co-design workshops will then be conducted with various stakeholders in two regions (Rhineland-Palatinate and Saxony) of Germany. Following the protocol proposed by the Stanford Design Thinking School, we will use design thinking to collect ideas for the intervention. Based on these ideas and our previous qualitative and quantitative study, we will develop an intervention in collaboration with didactic experts. The intervention will be piloted in three groups (midwives=10–15, midwives working as practice supervisors=5–10, students of midwifery=10–20). For the process evaluation, we will use observation protocols of the intervention conduct and qualitative interviews. For the outcome evaluation, we will use a questionnaire and observations in simulation laboratories with students of midwifery.

**Ethics and dissemination:**

This study protocol was approved by the Ethics Committee of the University of Regensburg (ID 23-3441-101) and is in compliance with the Declaration of Helsinki. Participation in the study will only be possible after informed consent has been given. Our results will be presented at national and international conferences and published in scientific journals. Additionally, once it has been finalised, we will make the intervention available to educational institutions for (future) midwives.

**STRENGTHS AND LIMITATIONS OF THIS STUDY**

- The target group participates in all steps of the intervention development; this ensures that the intervention is relevant, well received and feasible in a real-world setting.
- Following the Medical Research Council framework throughout the development process ensures high intervention quality.
- Our mixed-methods approach provides us with a thorough understanding of the context and the needs of our target group.
- Testing the intervention in different target groups provides early insights into feasibility and preliminary outcomes, which can help refine the intervention prior to a larger trial.
- The intervention will only be developed in two regions in Germany, and we may therefore not be able to consider all regional differences.

## Introduction

 The study is based on the increasing recognition of health literacy (HL) as an important factor influencing health outcomes. Since 2020, HL using early childhood allergy prevention (ECAP) has been the focus of the Health Literacy in Early Childhood Allergy Prevention (HELICAP) research group. HL is understood as the knowledge, motivation and competence to access, understand, appraise and apply health information. This does not, however, rely solely on the skills of the individual, but is located in a broader context and affected by a range of determinants including situational, societal and environmental.^1^

Studies show that low HL is associated with greater use of the healthcare system,^2 3^ less healthy nutrition, less exercise and poorer dental hygiene,^4^ as well as a lower level of adherence to recommendations.^5^ This also applies to preventive behaviour, such as in the case of allergies. The incidence of allergies is increasing worldwide, affecting well-being and even threatening life (anaphylaxis).^6^,^8^ Research indicates that families can lower their children’s risk of developing allergies by adopting certain health behaviours during the first 1000 days of life.^8^,^10^ Health professionals, especially midwives, are important sources of information on health-related topics, such as infant feeding and hygiene. They can therefore have a significant impact on ECAP for expectant and new parents.^11 12^ However, for health professionals to effectively communicate recommendations, they have to understand the scientific evidence on which they are based. In the case of allergy prevention, there has been a significant shift in evidence, indicating that early exposure rather than avoidance of allergens may reduce the risk of developing allergies. To date, however, the evidence remains uncertain and inconsistent.^9 13^ This presents a challenge, making this example a particularly interesting case for HL research.

The planned study will build on the results of our previous work.^14^ We found that paediatricians and midwives rarely used HL-responsive techniques in consultations on ECAP with parents.^15 16^ Midwives also lacked knowledge about ECAP and HL. This may lead to an overestimation of parental understanding of health-related information regarding ECAP.^17^

In Germany, postnatal care by midwives is widely provided in the family’s home. After giving birth, mothers are entitled up to two home visits a day for the first 10 days, followed by a further 16 visits by the midwife during the first 12 weeks, and 8 more up until the end of the ninth month.^18^ This is a unique consultation context as midwives are offering advice in the intimate home environment, enabling them to obtain a more holistic impression of the family and their way of life. This access to families provides unique opportunities to offer health-related advice. To date, the role of this consultation context in relation to the provision of HL-responsive advice on ECAP as well as other topics by midwives has been understudied—a research gap that needs to be addressed. In our understanding, HL-responsive advice comprises the communication of evidence-based health information in a way that enables people to understand, appraise and apply this information with a view to engaging and supporting them in making health-related decisions.^19 20^

Educational interventions on providing HL-responsive advice can improve communication between health professionals and patients.^21^,^24^ Studies on clear bedside communication have shown that a curriculum consisting of didactic and interactive workshops can improve knowledge, skills and attitudes.^21 22^ The Health Literacy Universal Precautions Toolkit for healthcare providers offers evidence-based guidance to promote better understanding aimed at all patients.^25^ However, different strategies are required in different cultures, settings and professions.^26 27^ To our knowledge, HL training for midwives has not been specifically addressed so far.^28^ Since 2020, midwifery in Germany requires a university education and is now offered as a dual Bachelor’s degree, combining classroom instruction and a practical placement. Germany therefore currently has midwives who were trained in vocational schools (until 2020), midwives who were trained in vocational schools and are now working as practice supervisors (a midwife who has obtained an additional teaching qualification enabling her to provide structured guidance, supervision and assessment of midwifery students during their practical training. This role requires both professional experience and formal training in midwife education) for future midwives studying midwifery, and midwifery students who are in the process of completing a university degree. The academic education of midwives now combines at least 2200 hours of theoretical instruction with 2200 hours of clinical practice. Clinical placements are undertaken in collaboration with hospitals and community-based midwifery practices, under structured clinical supervision. In addition, simulation laboratories are a compulsory part of the midwifery curriculum, serving as a bridge between classroom teaching and clinical placements by providing a safe environment in which to practise and demonstrate counselling skills.

The aim of this study is to develop an intervention for midwives in a co-design process, addressing the different groups of midwives described above. This intervention will enable midwives to (a) consider and assess parental HL when advising on ECAP, (b) advise parents in an HL-responsive way and (c) support HL in families in general. By employing a co-design approach that involves stakeholders in the development process, the study seeks to create a relevant and practical intervention focusing on the needs and preferences of the target group.

## Methods and analysis

### Study design

**Figure 1:**
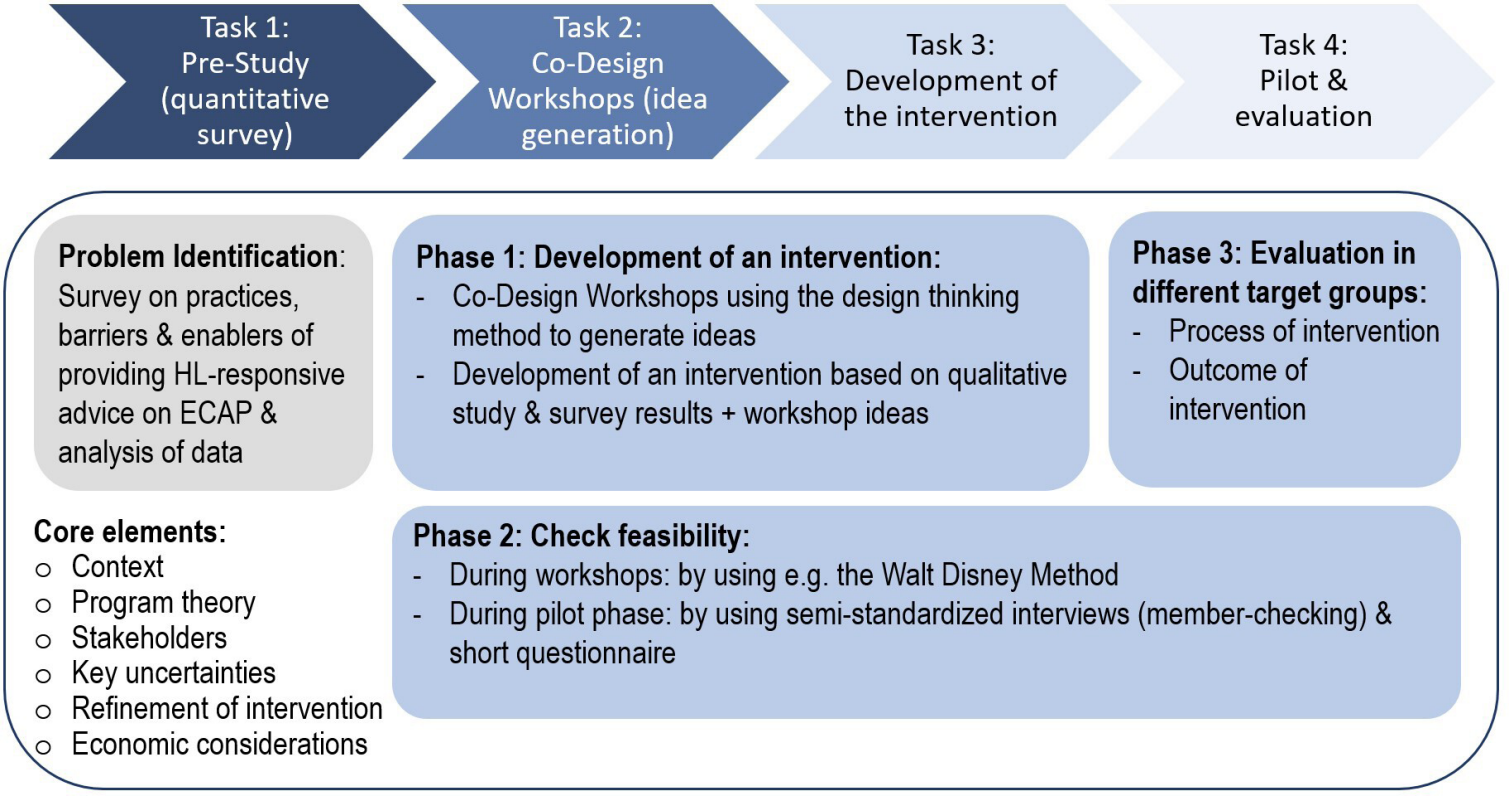
Flowchart on study design and anticipated tasks based on the MRC framework. ECAP, early childhood allergy prevention; HL, health literacy; MRC, Medical Research Council.

After thoroughly exploring the context (problem identification, see figure 1: Task 1), we will follow the Medical Research Council (MRC) framework for developing and evaluating complex interventions.^29 30^ This framework seemed highly suitable for our research as it is well known and well established and focuses on the development and evaluation of complex interventions in the clinical context. The updated framework from 2024 pays increased attention to the core element of ‘context’, which we consider highly important for midwifery care, as this differs significantly both between countries and within Germany. The framework suggests four phases for intervention research, with each phase having a common set of core elements (context, programme theory, stakeholders, key uncertainties, refinement of intervention and economic considerations; see figure 1). We will focus on three of the phases outlined in the MRC framework: development of the intervention, feasibility and evaluation. The implementation phase will not be part of the current study. We used the guideline for reporting intervention development studies (GUIDED) for this manuscript (see online supplemental appendix 1).

### Task 1. Pre-study (quantitative survey): problem identification

We will conduct an exploratory quantitative study, using an online survey to obtain representative data on how midwives provide HL-responsive advice on ECAP. This will offer insights into the context and a starting point for the development of the intervention, focusing on the specific needs of midwives (problem identification, see figure 1: Task 1). The survey aims to quantify midwives’ knowledge, skills, attitudes and practices, as well as to identify barriers and enablers when it comes to providing advice on ECAP in an HL-responsive way. We will also collect data on the needs and wishes of the respondents when it comes to providing advice, as well as certain sociodemographic characteristics of the respondents.

After performing a literature review, we developed the questionnaire based on the results of a previously conducted qualitative study exploring how midwives provide advice on ECAP and the extent to which they take parental HL into account when doing so.^15^ We extracted items regarding current practices from these qualitative results. To develop items regarding barriers and enablers, we used the Cabana framework,^31^ which focuses on barriers regarding adherence to clinical practice guidelines and the Theoretical Domains Framework,^32^ which concentrates on identifying determinants of current and desired professional behaviour. We applied both frameworks to our qualitative results, identifying the categories that were most relevant to our research (see online supplemental appendix 2).

In an iterative process involving discussions with experts (midwives and public health experts with experience in questionnaire development), we formulated items based on these categories.

The questionnaire consists of closed-ended questions with single (yes/no) or 5-point Likert-type items, multiple-choice items and open-ended questions.

To ensure the content validity of the questionnaire, we conducted cognitive interviews with midwives (n=5) and experts in ECAP and HL (n=3) using the think-aloud technique and intensive probing.^33^ The interviews lasted 45 min on average. They were audio-recorded and transcribed and their content was analysed. Instructions, questions and response categories were mostly rated as easy to understand, with some minor changes required regarding the wording. Some questions made the participants reflect on their own professional practice, which suggests the relevance of the items. Answer options were considered appropriate. Completeness was mostly rated as achieved. After adapting the questionnaire, we piloted it in Saarland and Berlin with n=59 midwives. Acceptability and comprehensibility also appeared to have been achieved as most questionnaires (n=52) were completed and we received no further questions or negative feedback.^34^

We calculated the mean and standard deviation (SD) and examined the response distributions for floor/ceiling effects and response bias patterns. For most items, the response options were fully used and distribution was acceptable. Two items showed a ceiling effect (topics considered important during the provision of advice on ECAP): with regard to ECAP, breastfeeding was considered important by almost all midwives, as was smoking; however, we decided to keep these items in the questionnaire to provide a complete picture of topics. Two items focusing on how advice on ECAP is provided during consultations were designed to be mutually exclusive; however, cross-tabulation showed that this was not the case. These items were therefore rephrased. To check internal consistency, we calculated Cronbach’s alpha for the following constructs: importance of topics in consultations on ECAP (11 items, 5-point Likert scale, α=0.81), professional HL regarding ECAP (four items, 5-point Likert scale, α=0.78), HL-responsive advice (seven items, 5-point Likert scale, α=0.77), perception of midwifery work in regard to providing advice on ECAP in an HL-responsive way (eight items, 5-point Likert scale, α=0.76). After minor adaptations, the 70-item questionnaire was finalised. The results of the questionnaire design and validation are in the process of being published.

#### Data collection

We will invite midwives working in antenatal and postnatal care from all German federal states to participate in our online survey. For the participant recruitment, we will draw on the steps of ‘INTACT-RS’, a theoretical framework developed by the HELICAP research group to guide recruitment from the perspective of potential study participants.^35^ Possible barriers to and enablers of recruitment can thereby be considered and addressed a priori. To encourage participation, we will create different formats to approach midwives, for example, personal emails, calls for participation on websites and posts on Instagram and LinkedIn. We will address the specific needs and concerns of potential respondents, for example, by highlighting the fact that we are interested in daily practice and that no preparation is necessary to participate. We will contact regional midwifery associations and use social media and snowball sampling. To increase the response rate, follow-up emails and posts will be used to remind the target group of the survey and its importance. We calculated our required sample size using an online sample size calculator.^36^ With 27 000 midwives as eligible participants in Germany,^37^ we require a sample of 379 to obtain a 95% CI and a 5% margin of error to achieve representative insights.

#### Data analysis

The quantitative data will be analysed by SPSS (IBM Corp. Released 2019. IBM SPSS Statistics for Windows, V.29.0.2.0, Armonk, New York: IBM Corp) using descriptive statistics. We will calculate means and SD for continuous data and frequencies and proportions for categorical data.

### Task 2. Co-design workshops: idea generation

**Figure 2:**
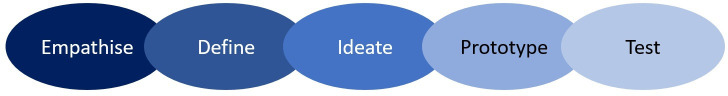
Anticipated co-design workshop flow focusing on idea generation.

In order to meet the needs of the target group and create an acceptable and sustainable intervention, it is necessary to engage them in the development process.^29^ We will conduct two co-design workshops, each with a duration of 7 hours, in two different locations in Germany (Mainz, Rhineland-Palatinate, July 2025, and Leipzig, Saxony, October 2025), with different stakeholders. The aim of the workshops is to generate initial ideas for an intervention on providing advice on ECAP in an HL-responsive way, looking at different educational formats and content (see figure 1, Phase 1: developing an intervention). The co-design workshops will be based on the design thinking method and modified for our specific aim.^38^,^40^ This ensures the needs of the target group are taken into consideration when creating a new intervention and those needs and the feedback provided are incorporated throughout the development process.^38^ Design thinking is a human-centred, iterative approach to problem identification and problem solving focused on the needs of the target groups. It has been used in different healthcare settings and conditions.^38^ The approach seems especially well suited to our aim of co-designing interventions with midwives, as it will enable us to actively engage them and learn about their specific needs. The creative approach of designing a prototype helps to crystallise ideas and further engage the participants. The steps of the Stanford Design School’s protocol for design thinking are (1) empathising with the target group, (2) defining the problem, (3) ideating, (4) prototyping and (5) testing. These steps will be followed during the workshop (see figure 2).^41^

#### Sample

We will invite a heterogeneous group of midwives and interdisciplinary experts on ECAP to ensure maximum expertise and experience and to involve all stakeholders that the intervention seeks to address (see table 1).

**Table 1:** Overview of workshop participants

Participants	Number per location (n=2)	Sample description
Midwives	n=4	Working in prenatal and postnatal care, >5 years’ professional experience
Students of midwifery	n=4	Studying midwifery, >2 years of studies
Midwives (practice supervisors)	n=4	Working as practice supervisor or teacher in midwife education, >2 years’ teaching experience
Interdisciplinary/interprofessional experts (e.g. on didactics or psychology)	n=4	Education or professional expertise in other scientific/professional fields
Experts on ECAP	n=3	Background or professional expertise in allergology

#### Recruitment

Midwives will be recruited via our existing contacts, established during our previous study, such as the German Midwives Association.^14^ In order to take into account the different perspectives on teaching, we will invite midwives working in antenatal and postnatal care, as well as those who are actively involved in the training of midwives (practice supervisors) and midwifery students to attend our workshops. Recruitment will be based on a purposive sampling strategy to ensure the participation of highly interested, knowledgeable and experienced individuals.^42^ Midwifery students will be recruited with the help of midwifery lecturers/professors. The focus will be on students in their fifth/sixth semester, as they will already have completed some of their studies and will also have had practical experience working as a midwife. Didactic and ECAP experts will be recruited based on existing connections. Each participant will be offered a remuneration of €100.

#### Workshop preparation

A didactic workshop concept will be developed based on the design thinking method mentioned above (see figure 2).

As our participants may not be familiar with HL and ECAP, we will prepare materials to help them relate to the topics. Regarding ECAP, we will draw on the results of previous studies conducted within the HELICAP research group^43 44^ and take into account the German Allergy Prevention Guideline as the most recent (2022) scientific source.^9^ Regarding HL, the holistic model developed by Sørensen *et al*^1^ will serve as a basis along with a consensus study on HL practices and educational competencies for health professionals.^45^ To provide the participants with information on the findings of the HELICAP group, we will draw on the materials developed during the first funding phase.^46^ These include brochures, podcasts and factsheets. Participants will be invited to review these documents prior to their participation. We will also share the results of our qualitative and quantitative study with them, offering additional insights into the practices, barriers and facilitators when it comes to providing advice on ECAP in an HL-responsive way, as well as midwives’ needs and wishes regarding further training in this area.

#### Workshop organisation

After introducing the design thinking process, the research team will provide the participants with information about ECAP, HL and the results of our qualitative and quantitative study,^15^ thus enabling the group to define the focus, scope and objectives of the intervention. Learning objectives for ECAP, HL and HL-responsive techniques will be developed jointly. In addition, different didactic methods will be used to provide inspiration for the intervention, for example, role play models, online learning tools, observational learning, inquiry-based learning and game-based learning.

The participants will then work collaboratively in small groups of four to five people. Each group will consist of different types of participant, for example, one midwifery student, one practice supervisor, etc. (see table 1). In order to empathise with the specific needs of a particular group of midwives, for example, students or practice supervisors, we will use different methods, for example, interviews and the A-E-I-O-U method (*empathise*). This is the starting point for defining the specific needs that the groups will address (*define*). They will then start to develop ideas, with no restrictions regarding feasibility or economic considerations, with the help of the Walt Disney or 6-5-3 method. At the end of this phase, the groups will be asked to prioritise their favourite idea (*ideate*).

In the next phase of the design thinking process, the participants will create a prototype of their favourite idea. They will be supplied with different materials (from a storyboard wall to LEGO) enabling them to use creative and hands-on approaches to visualise and turn their ideas into reality (*prototype*). At this stage, the main uncertainties about the proposed intervention ideas will be identified and the focus will shift to feasibility (see figure 1).

Finally, each group will present their prototype to the other groups (*test*). All participants will be invited to provide feedback and ideas on how to enhance the prototype presented.

The workshops will be audio-recorded and transcribed. The research team conducting the workshop will closely follow the process and create an observation protocol. The focus of the protocol will be on group interaction. The aim is to be able to track ideas that were either not considered at all or rejected during the development process and understand why this was the case, for example, due to group dynamics. These ideas may still be interesting and provide additional insight into specific needs.

### Task 3. Intervention development

In the next step, the research team will focus on the development of a programme theory aimed at specifying how the intervention is meant to lead to its effects and under what conditions. A logic model focusing on resources/inputs, activities, outputs and outcomes will be prepared to theoretically establish how the intervention is expected and intended to work.^26 47^

The research team will jointly review all documents and prototypes created during the workshops. Drawing on the results of the qualitative study of midwives conducted previously^15^ and the Germany-wide online survey, the research team will work with the didactic experts, will focus on the ideas the workshop participants deemed most relevant and valuable and combine them into a coherent intervention. Midwives are accustomed to working in a problem-oriented context. We assume that the central component of the intervention will be educational and will follow the principle of problem-based learning.^48^

The documents needed to deliver the intervention will be developed and prepared by the research team. These might include case studies, practical instructions and information leaflets, with different versions for the different intervention contexts (students, practising midwives, midwives working as practice supervisors). Once these documents have been finalised, they will be shared with and reviewed by the workshop participants. Participants will be invited to an online workshop where they will discuss and provide feedback on (a) necessary improvements and (b) ideas on where and how to pilot the intervention. Different workshop timeslots will be offered to the participants to enable as many as possible to provide feedback on the final intervention.

The format, content and materials are intended to be developed as (a) module(s) for (further) training, or a ‘toolbox’ or ‘toolkit’ to be used by educational institutions for midwives, midwifery students and (in future) target groups in the healthcare field who also provide advice on health topics.

### Task 4. Intervention piloting and evaluation

The intervention will be piloted in relevant settings. Prior to the piloting, we will develop an evaluation plan based on our programme theory. This will include various steps enabling us to focus on the process and outcome in order to evaluate and subsequently further improve and adapt the intervention, if necessary.^26 27 29^ Changes may be made to our piloting and evaluation plan depending on the intervention developed.

#### Sample and recruitment

We anticipate the intervention being conducted either by someone from the research team or someone who is involved in the (further) training and education of midwives. The intervention will be conducted with (a) midwives (n=15–20), (b) midwives working as practice supervisors (n=5–10) and (c) students of midwifery (n=10–20). Sample sizes may be adapted if needed. Participants from groups (a) and (b) will once again be recruited via the German Midwives Association, personal contacts and snowballing. The implementation of the intervention within midwifery degree courses will take place at the University of Mainz. All participants will be asked to evaluate the intervention.

#### Data collection and analysis

##### Process evaluation

In order to gain insights into the process of conducting the intervention, one of the research team will either carry out the intervention themselves or be present and closely observe (with the use of observation protocols) the implementation of the intervention by a third party, for example, from an educational institution. Semistructured interviews will be carried out with the individuals involved in conducting the intervention in order to learn more about their experiences. The focus will be on barriers and facilitators, as well as the feasibility of the intervention.

Furthermore, the intervention will be evaluated with a focus on the perspective of the participants receiving the intervention.^49^ They will be invited to take part in short semistandardised interviews immediately after the intervention. The aim is to conduct interviews with at least three participants from each group (midwives, midwives working as practice supervisors, midwifery students). The interviews will focus on:

- Overall perception of the intervention: Is it appreciated? Are the didactic methods helpful? Is the information provided new/considered useful?
- Transfer to practice: Can the learning content (ie, HL and ECAP) be put into practice? Is there anything missing that would assist with that process?
- Possibilities for improvement: How can the intervention be improved in terms of its implementation, provision of materials, etc.?

Further questions will be developed once the intervention has been finalised.

All interviews will be audio-recorded, transcribed and subjected to content analysis performed by two researchers and a student assistant.^50^ The results of the interviews will be triangulated with the observation protocols.

##### Outcome evaluation

To evaluate the outcome of the intervention, we will develop a short version of the questionnaire on providing HL-responsive advice. We will do this once the focus of the intervention has been established and its development completed to ensure the changes brought about by the intervention can be measured. Participants receiving the intervention will be asked to complete the questionnaire before and then again three months after the intervention. The quantitative data will be analysed using descriptive statistics. The means and SD will be calculated for continuous data and frequencies and proportions for categorical data. We will compare the results from before and after the intervention to establish whether the intervention enhanced the recipients’ knowledge and skills.

In addition, within the subgroup of midwifery students at the University of Mainz, we will assess the intervention’s effects on knowledge and competence regarding the provision of advice on ECAP in an HL-responsive way. Therefore, half of the participating students will receive their training based on the intervention developed and the other half of the students will receive their training according to the existing curriculum. Subsequently, all students will engage in simulated consultation sessions on ECAP. The students will be given the task of providing advice on ECAP to a family (played by actors) in the simulation laboratories. The latter provide a controlled environment in which to practise consultations in realistic but standardised scenarios. The knowledge and proficiency levels of the members of both groups will be evaluated according to a predefined checklist (objective structured clinical examination). This checklist includes the horizons of expectations regarding ECAP in a consultation situation, as well as the corresponding grading schemes that are commonly used. The controlled and standardised environment of the simulation laboratory will enable us to gain a thorough understanding of the differences in knowledge and consultation skills between students who have received the intervention and those who have not.

Based on the results of the process and outcome evaluations, the intervention will be adapted if necessary.

### Study status

During the process of publishing the present protocol manuscript, tasks 1 and 2 have commenced. Data were collected via the online survey from November 2024 to January 2025. The analysis of the data is currently ongoing. The co-design workshops were conducted in July and October 2025. Intervention development will begin in November 2025. The piloting and evaluation of the intervention are expected to begin in April 2026. The project is scheduled to conclude in May 2027.

### Patient and public involvement

The involvement of the target group is a core element of our study. The research team consists of two midwives. The ideas generated by midwives and other participants will benefit the development of the intervention, ensuring the midwives’ point of view features in every step of the intervention development process. We used the Guidance for Reporting Involvement of Patients and the Public (GRIPP2) checklist to indicate patient and public involvement in our study (see online supplemental appendix 3).^51^

## Ethics and dissemination

### Ethics approval and consent to participate

This study was approved by the Ethics Committee of the University of Regensburg (ID 23-3441-101). The study conforms to the Declaration of Helsinki.^52^

The data to be collected within this study will consist of anonymous survey data, workshop protocols, qualitative data from evaluation interviews (audio recordings and transcripts) and questionnaires from participants in the intervention. Participation in the study will only be possible after informed consent has been given.

### Storage and technical archiving

Data will be entered into SPSS and Atlas.ti for analysis. Personal identifiers will be removed early in the data collection process. All databases and software used (SPSS 28, Atlas.ti 8) are stored on secure servers at the University of Magdeburg. The PCs and data carriers used will be protected by the personal passwords of the project staff in order to prevent any access to the data by third parties in the absence of the project staff. Audio tapes, transcripts, minutes and notes will be kept in a secure, locked location to which only authorised persons will have access. A numbered hard copy of the protocols and transcriptions will be retained, as this will enable easy follow-up in the case of questions. An additional electronic copy will also be stored to be used for data confirmation and/or audits. In accordance with good scientific practice, the research data will be archived for at least 10 years in the Institute of Social Medicine and Health System Research, Magdeburg.

### Legal obligations and conditions

All persons involved in the collection, transcription and analysis of data will be trained in data collection procedures and the protection of participants’ privacy and confidentiality and will be bound by the Data Protection Act (DS-GVO).

### Enabling subsequent reuse and long-term accessibility

The data collected will be used for scientific purposes only and will not be shared with third parties. The results of this study will be published in peer-reviewed open-access journals. Findings will also be presented at relevant conferences. The online survey data, workshop protocols and interview transcripts may be made available to the scientific community in anonymised form via electronic research data repositories (such as GESIS or Dryad) in the medical and social sciences.

## Discussion

Our study builds on the findings gathered within the HELICAP research group starting with a summary of the existing scientific evidence on ECAP, how it is translated into national guidelines and the extent to which parents adopt HL behaviours during pregnancy and early childhood.^1244 53^,^55^ Together with this background information, the qualitative findings from our previous study with health professionals and our quantitative results of a survey focusing on midwives will provide a sound basis for the development of an intervention, providing data on regional differences, differences between target groups, etc. ^15 16^

We also expect that involving the target group in the development of interventions leads to better results in terms of acceptability and sustainability.^56^ We aim to involve our target group in all the steps of intervention development, from idea generation to the piloting of the intervention. Collecting participants’ feedback on the intervention will enable the research team to make the necessary adjustments and improvements based on participant experiences and suggestions. Previous research on intervention development shows that it is paramount to gain a thorough understanding of the context and the target group in which the intervention will be applied.^57^

We therefore anticipate the development of an intervention to support midwives in providing HL-responsive advice on ECAP and other topics, which will be widely used in midwifery studies and further training. Findings from this intervention will not only contribute to the existing body of knowledge on HL in midwifery but also inform future training programmes and policies aimed at improving health communication in healthcare settings. The anticipated outcomes of the intervention include improved communication between midwives and parents, leading to better adherence to health recommendations and ultimately improved child health outcomes. In time, these efforts will lead to HL-responsive consultations by midwives, which assess and are mindful of parental HL when providing advice on ECAP and other health-related topics.

The intervention is developed based on workshops conducted in only two regions in Germany, which may pose a limitation to the study. It is possible that additional regional variations may exist that have not been identified. A larger implementation, which is not part of this study, may provide further insights and enhance the intervention in the future.

## Supplementary material

10.1136/bmjopen-2024-098402 online supplemental file 1

10.1136/bmjopen-2024-098402 online supplemental file 2

10.1136/bmjopen-2024-098402 online supplemental file 3
