# Supplementary material for: How can midwives in Germany be supported in advising on early childhood allergy prevention in a health literacy-responsive way? Protocol for a mixed-methods study to co-design and evaluate an educational intervention following the Medical Research Council framework
Source: BMJ Open. 2025 Dec 11;15(12):e098402. doi: 10.1136/bmjopen-2024-098402 (PMC12699726; doi:10.1136/bmjopen-2024-098402)
Supplement: online supplemental file 2 [file bmjopen-15-12-s002.docx]

| **Categories** | **Cabana Framework** | **Theoretical Domains Framework** |
| --- | --- | --- |
|  | | |
| 1.) Practice of HL-sensitive counselling on ECAP | | |
| 1.1.) Assessment of allergy risk | | |
| 1.2.) Topics considered important when providing advice on allergies | | |
|  | | |
| 2.) Barriers & Enablers of HL-sensitive ECAP counselling | | |
| 2.1.) Lack of Awareness, Lack of Familiarity / Knowledge | x | x |
| 2.2.) Lack of Agreement | x |  |
| *Lack of Self-Efficacy* | x |  |
| *Beliefs about capabilities* |  | x |
| 2.3.) Lack of Outcome Expectancy / Beliefs about Consequences | x | x |
| *Inertia of Previous Behaviour* |  | x |
| *Nature of behaviours* |  | x |
| 2.4.) External barriers / Environmental Context and resources, Social influences | x | x |
| 2.5.) Skills |  | x |
| 2.6.) Social Role and Identity |  | x |
| 2.7.) Motivation |  | x |
| *Memory, attention and decision processes* |  | x |
| *Emotion* |  | x |
| *Behavioural regulation* |  | x |
|  | | |
| 3.) Needs & Wishes | | |
| 4.) Sociodemographic data | | |

*Appendix 2: This is an overview of the overarching categories (grey background) that served as basis for the item development and from which framework (Cabana Framework and Theoretical Domains Framework) they were derived. The categories in italic / light grey were excluded or subsumed.*
