# Supplementary material for: How can midwives in Germany be supported in advising on early childhood allergy prevention in a health literacy-responsive way? Protocol for a mixed-methods study to co-design and evaluate an educational intervention following the Medical Research Council framework
Source: BMJ Open. 2025 Dec 11;15(12):e098402. doi: 10.1136/bmjopen-2024-098402 (PMC12699726; doi:10.1136/bmjopen-2024-098402)
Supplement: online supplemental file 3 [file bmjopen-15-12-s003.docx]

| **Section and topic** | **Item** | **Reported on page No** |
| --- | --- | --- |
| 1: Aim | Report the aim of PPI in the study | P. 3 |
| 2: Methods | Provide a clear description of the methods used for PPI in the study | P. 5-8 |
| 3: Study results | Outcomes—Report the results of PPI in the study, including both positive and negative outcomes | P. 8-9 |
| 4: Discussion and conclusions | Outcomes—Comment on the extent to which PPI influenced the study overall. Describe positive and negative effects | P. 10 |
| 5: Reflections/critical perspective | Comment critically on the study, reflecting on the things that went well and those that did not, so others can learn from this experience | n/a (study protocol) |

*Appendix 3: patient and public involvement (PPI) following the GRIPP 2 short form*
