# Supplementary material for: How can midwives in Germany be supported in advising on early childhood allergy prevention in a health literacy-responsive way? Protocol for a mixed-methods study to co-design and evaluate an educational intervention following the Medical Research Council framework
Source: BMJ Open. 2025 Dec 11;15(12):e098402. doi: 10.1136/bmjopen-2024-098402 (PMC12699726; doi:10.1136/bmjopen-2024-098402)
Supplement: Supplementary file 4 [file bmjopen-2024-098402.reviewer_comments.pdf]

PEER REVIEW HISTORY

BMJ Open publishes all reviews undertaken for accepted manuscripts. Reviewers are asked to complete a checklist review form and are provided with free text boxes to elaborate on their assessment. These free text comments are reproduced below.

ARTICLE DETAILS

Title (Provisional)

How can midwives in Germany be supported in advising on early childhood allergy prevention in a health literacy-responsive way? Protocol for a mixed-methods study to co-design and evaluate an educational intervention following the Medical Research Council framework

Authors

von Sommoggy, Julia; Steinmann, Julia Ruth; Lander, Jonas; Bitzer, Eva Maria; Pawellek, Maja; Brandstetter, Susanne; Apfelbacher, Christian; Fillenberg, Barbara Dorothea

---

VERSION 1 - REVIEW

**Reviewer**	**1**
**Name**	**Cheng, Christina**
**Affiliation**	**La Trobe University**
**Date**	**07-Feb-2025**
**COI**	**None**

This is an interesting study, and the description of the protocol is generally good, but some details need to be clarified. A main issue is language, there are terms or words that are not commonly used in English. A good editing and proofreading of the manuscript are needed. Besides, the dates of the study need to be included in the manuscript as per journal's guidelines. Here are my comments:

Major comments:

1. Abstract – Methods & Analysis section, you have not mentioned the design thinking method that you'll be using.

2. Strengths and limitations of this study – the fourth dot point is about your evaluation method; they are not exactly 'strengths' or 'limitations'.

3. Study Design – Please provide the rationale for choosing this framework. Have you incorporated the recently revised framework:
https://www.sciencedirect.com/science/article/pii/S0020748924000609#s0020

4. Study Design – Lines 119-127, this section of 'The tasks will be…' is a replication of the following sections.

5. Study Design – How was your questionnaire developed? How can you ensure that you had covered all potential barriers or enablers? Your Introduction indicated that midwives lack knowledge of HL and HL-sensitive ECAP counselling based on your previous research, will there be items in the questionnaire to help determine participants' HL and HL-sensitive counselling knowledge? How will these be measured? Can you attach the questionnaire as supplemental materials? Without the questionnaire, it is difficult to consider if you have collected the right type of data. Alternatively, you can also provide a full list of your variables.

6. Study Design – How was the content validation of the questionnaire done?

7. Study Design – Line 149, you mentioned 'tailored materials', can you attach some 'tailored materials' as examples as supplemental materials to demonstrate what you mean by 'tailored' materials?

8. Study Design – Line 200, can you provide a rationale for using the Design Thinking method, this method is 'borrowed' from design and mostly used for business. There are many other co-design processes/frameworks in health. Why use this one? You will see below that I also have some further comments about the process.

9. Study Design – Lines 226-227, 'the groups will conduct an interview with a member of another group' This is confusing. You have 18 participants, please first lay out how many groups are planned, how to determine which participant go to which group. With this sentence, do you mean some members of these groups will interview each other?

10. Study Design – Line 288, Empathize is an important element of the Design Thinking. It should involve observe, engage, watch and listen. I don't think just talking to someone within the timeframe of a session in a workshop allows enough time to observe, engage, watch and listen to fully complete the 'Empathize' process.

11. Study Design – Line 233 'protocols'. When you mentioned protocols as in 'document the process in detailed protocols, focusing on the ideas generated', it seems to refer to the process of your workshop. Here, the 'protocols' will then be edited… seems to refer to the protocol of the intervention. Please clarify. Also, does it mean that the ideate process will not only generate an idea but also the protocol?

12. Study Design – Line 234, 'completion'. What does it mean by 'completion'? Who will decide if an idea is 'complete' or not? I think the prototype is the next workshop?

13. Study Design – Line 241 'discussed in plenary'. Does it mean there'll be small groups again?

14. Study Design – How long will workshop 1 or 2 be? Is discussion of both workshops recorded and transcribed?

15. Study Design – Workshop 2. The 'Prototype' and 'Test' of the Design Thinking method used in workshop 2 will have different meaning to health intervention. How do you adapt these steps to the health context?

16. Study Design – Line 249-251. The core element 'programme theory' of the MRC framework is more than a logic model, it also identifies the mechanism: 'Programme theory describes how an intervention is expected to lead to its effects and under what conditions. It articulates the key components of the intervention and how they interact, the mechanisms of the intervention, the features of the context that are expected to influence those mechanisms, and how those mechanisms might influence the context.'

17. Study Design – Line 253, 'qualitative study with midwives'. Are you referring to the study from your previous work?

18. Study Design – Line 257, 'will be educational…' Would such an assumption affect your choice of intervention for development?

19. Study Design – Line 263, 'online workshop'. Would this be workshop 3? Given there are 3 workshops, how will you handle dropouts?

20. Study Design – Line 280, sample size of intervention. Is there any rationale for the sample size?

21. Study Design – Line 280, what is the difference between participants a (in practice) and participants b (as practice guide)?

22. Study Design – Line 289, about delivery of intervention. As the participant deliver the intervention, will someone be at the receiving end of the intervention? How will you recruit people who's on the receiving end? Will these people on the receiving end also be interviewed?

23. Study Design – Line 290, interviews. How many interviews are planned?

24. Study Design – Line 294, 'member-checking'. What do you mean by 'member-checking'? Do you mean people receiving the intervention?

25. Study Design – Line 296, 'interviews'. Which interview? I think you have proposed 2 interviews, one with the person delivering the intervention and another which I am not quite sure who you referred to.

26. Study Design – Lines 304-305 interview data. How many researchers will be involved in the analysis of the interview data?

27. Study Design – Line 310, quantitative data. The sample size is small, has your sample size calculation ensure that you have enough power to detect changes?

28. Study Design – Line 315, simulation lab. What is the purpose of this additional testing? The description isn't too clear about the procedure. What does it mean that only one group

will participate in the educational intervention? Do you mean there'll be an intervention group and a control group? How many participants are you expecting?

29. Study Design – Do you think your evaluation needs to be adjusted when you have decided on the intervention?

30. Study Design – Line 323, the sentence is incomplete.

31. Patient and Public Involvement Statement – I think you can also mention the workshop participants (the midwives) as public involvement.

Minor comments:

1. Target group vs target groups – Throughout the manuscript, you have 'target group' and you have 'target groups'. I think you have midwives as your target group, do you also have other target groups? It is confusing.

2. Background – First paragraph, line 66, the term 'use case', I am not sure what you mean by 'use case'.

3. Background – First paragraph, lines 66-69, the sentence is too long and difficult to read.

4. Background – Second paragraph, line 74, please check how numbers are reported as per journal's guidelines.

5. Background – Lines 74-75, the sentence 'Research indicates…', the way this sentence is written makes me think that it is the newborn who should adopt certain health behaviours. I think you mean the mother needs to adopt certain health behaviours, is that correct? Please rewrite to clarify.

6. Study Design – line 112, 'cf. Figure 1', I'm not sure why you use 'cf.'. To ask readers to see a figure, you can simply say 'see Figure 1'. Besides, Figure 1 is your study process, not just problem identification, so, you need to make it clear like '(Problem Identification, see Task 1 of Figure 1)'.

7. Study Design – Lines 134-135, the term 'representative insight' is not a term commonly used in English even though I know what you mean.

8. Study Design – the '70-items questionnaire' should be '70-item questionnaire'.

9. Study Design – Line 164 the subheading of Task 2, I'd suggest using the same wording as in Figure 1.

10. Study Design – Lines 216 and 236, description of the workshops, I'd suggest just use 'Conducting of co-design workshop 1 (Idea generation), then, 'Conducting of co-design workshop 2 (concretization). Try not to create too many different terms for your co-design workshops.

11. Study Design – Line 282, 'conduction'. I don't think this is the right word. Do you mean 'implementation'? You have used 'conduct' in other parts of the manuscript as well.

VERSION 1 - AUTHOR RESPONSE

Reviewer: 1
Dr. Christina Cheng, La Trobe University
Comments to the Author:
This is an interesting study, and the description of the protocol is generally good, but some details need to be clarified. A main issue is language; there are terms or words that are not commonly used in English. A good editing and proofreading of the manuscript are needed. Besides, the dates of the study need to be included in the manuscript as per journal's guidelines. Here are my comments:

Dear Dr. Christina Cheng, thank you very much for reviewing our manuscript so thoroughly. We appreciate your comments and your suggestions and believe our manuscript is significantly improved by them.
Besides many other changes, the manuscript was now reviewed by a German teacher and an Australian native speaker. Thus, English proficiency is given, as well as knowledge of the Germany language, which ensures, that translations are correct. We hope the manuscript now meets your expectations. The page and line references your find below refer to the version of the manuscript with tracked changes.

We included the current status with the respective dates:
P.6 Line 210-211: *This task has already commenced during the process of publishing this manuscript. Data is collected and currently being analyzed.*
P. Line: *We will conduct two co-design workshops with a duration of seven hours in two different locations (Mainz, Rhineland-Palatine July 2025 and Leipzig, Saxony October 2025) in Germany […]*

Major comments:
1. Abstract – Methods & Analysis section, you have not mentioned the design thinking method that you'll be using.

The design thinking method we are using stems from the Standford Desing School's Protocol for Desing Thinking as mentioned in Task 2: Co-Design workshops: idea generator P. 7, Line 230-231.

We included this now in the abstract as well
P. 1 Line 42: *Following the Stanford Design Thinking School's Protocol we will collect ideas for the intervention development employing the design thinking method.*

2. Strengths and limitations of this study – the fourth dot point is about your evaluation method; they are not exactly 'strengths' or 'limitations'.

We considered it a strength to use the simulation labs as quasi experiment, to really understand what changes may occur during consultations when future midwives are trained with our intervention compared to those, who are not trained with our intervention.

We rewrote this section to make this clear:

P. 2 Line 62: In simulation laboratories, we will perform a quasi-experiment: one group will be trained with our intervention; skills in providing advice will subsequently compared to another group without being trained with the intervention, but in the conventional way.

3. Study Design – Please provide the rationale for choosing this framework. Have you incorporated the recently revised framework: https://www.sciencedirect.com/science/article/pii/S0020748924000609#s0020

We chose the MRC Framework for developing complex interventions as it is a well-known framework, applied for many intervention developments in the clinical context.

We included a sentence in the study design section explaining the choice of framework:
P. 4 Line 125-127: This framework appears very suitable for our research as it is a well-known, well-established framework, focusing on the development and evaluation of complex interventions in the clinical context.

Thank you for pointing out this new publication, which we haven't considered so far. The study protocol is based on an application for a grant, which was positively evaluated in 2023, which is why we used the version from 2022 of the MRC framework. However, as the revised elements fit our study very well (paying closer attention to the core element 'context' for example) we included the updated version as a reference and added a sentence.

P. 4 Line 127-129: The updated framework from 2024 pays increased attention to the core element 'context' which we consider highly important in the context of midwifery care, as this differs significantly between countries, but also within Germany [28].

4. Study Design – Lines 119-127, this section of 'The tasks will be…' is a replication of the following sections.

Thank you for this remark. We deleted this section in the manuscript.

5. Study Design – How was your questionnaire developed? How can you ensure that you had covered all potential barriers or enablers? Your Introduction indicated that midwives lack knowledge of HL and HL-sensitive ECAP counselling based on your previous research, will there be items in the questionnaire to help determine participants' HL and HL-sensitive counselling knowledge? How will these be measured? Can you attach the questionnaire as supplemental materials? Without the questionnaire, it is difficult to consider if you have collected the right type of data. Alternatively, you can also provide a full list of your variables.

We were hoping the manuscript of the questionnaire development would be published by now, however your comment makes clear, that this section about the questionnaire needs further explanations. Thank you for pointing this out.

We rewrote and specified this section:
P. 5-6 Line 164-184: After performing a literature research, the questionnaire was developed based on the results of the previously conducted qualitative study exploring how midwives provide advice on ECAP and in how far they consider

parental HL [15]. We extracted items regarding current practices from these qualitative results. To develop items regarding barriers and facilitators, we used the Cabana Framework [30], which focuses on barriers regarding the adherence to clinical practice guidelines and the Theoretical Domains Framework [31], which focuses on identifying determinants of current and desired professional behaviour. We applied both frameworks to our qualitative results, identifying categories most relevant to our research.
In an iterative process involving discussions with experts (midwives and public health experts with experience in questionnaire development) we formulated items based on these categories.
To ensure content-validity of the questionnaire, we conducted cognitive interviews with midwives (n=5) and experts in ECAP and HL (n=3) using the think-aloud technique and intensive probing. The interviews lasted 45 minutes on average. They were audio-recorded, transcribed and content-analysed. Instructions, questions and response categories were mostly rated as easy to understand, with minor changes regarding the wording. Some questions made the participants reflect on their professional practice, which suggests the relevance of the items. Answer options were considered appropriate. Completeness was mostly rated as given. After adapting the questionnaire, we piloted it in Saarland and Berlin with n=59 midwives. Response options were mostly used in full. Acceptability and comprehensibility appear to be given as most questionnaires (n=52) were answered in full and we received no further questions or negative feedback.
After minor adaptations, the questionnaire was finalized.

Additionally we attached a file containing the categories of the questionnaire (see supplementary material).

6. Study Design – How was the content validation of the questionnaire done?

See above. We outlined the questionnaire development now extensively and explained more thoroughly how the content validation was done.

7. Study Design – Line 149, you mentioned 'tailored materials', can you attach some 'tailored materials' as examples as supplemental materials to demonstrate what you mean by 'tailored' materials?

Thank you for pointing out that this is not understandable. We rewrote this section and hope to make this clearer.
P. 6 Line 193-197: *To encourage participation, we will create different formats to approach midwives, e.g. personal e-mails, calls for participations on websites, posts on Instagram and LinkedIn and we will address the specific needs and concerns of potential respondents, e.g. highlighting the fact that we are interested in daily practices and no preparation is necessary to participate.*

8. Study Design – Line 200, can you provide a rationale for using the Design Thinking method, this method is 'borrowed' from design and mostly used for business. There are many other co-design processes/frameworks in health. Why use this one? You will see below that I also have some further comments about the process.

We agree with the reviewer, that there are many different co-design processes in health. We chose the design thinking method for various reasons. It seemed sensible to include different stakeholder (midwives, midwifery students and

midwives working as practice instructors in students education) as well as experts (psychology, didactics etc.), which is in integral part of design thinking. Furthermore, the framework of design thinking offers the opportunity to include different creative methods. As it is not our aim to develop an intervention in the workshop, but rather collect ideas for an intervention, we are not solely interested in the final result, the prototype. We are interested in the entire process of the workshops, from empathizing and understanding needs of midwives, to ideas generated visualized as a prototype. We believe that health professionals like midwives who are involved in practical care on a daily basis, are activated by being creative with their hands and appreciate the opportunity of design thinking to really design and visualize their ideas.

We included two sentences to convey the idea to the reader, however tried to keep it short, to not lengthen the study protocol too much.

P. 7 Line 225-230: *It is a human-centred, iterative approach to problem identification and problem solving for needs of target groups. It been used in different healthcare settings and conditions [35]. This approach seems especially suitable for our aim to co-design ideas for interventions with midwives, as we will be able to actively engage them as well as learn about their specific needs in this process. The creative approach of designing a prototype helps to concretize ideas and further engage the participants.*

9. Study Design – Lines 226-227, 'the groups will conduct an interview with a member of another group' This is confusing. You have 18 participants, please first lay out how many groups are planned, how to determine which participant go to which group. With this sentence, do you mean some members of these groups will interview each other?

Thank you for pointing this out. We realize this is a very confusing description of our approach. We rewrote the entire section regarding the conduct of the workshops. As we proceeded in the project since writing and submitting the study protocol, we are much more deeply involved in the method of design thinking and methods used within the design thinking method. We also conducted a pilot workshop in the meantime and learned a lot. We thus decided to adapt the conduct of the workshops, which is why we rewrote the entire section now. We hope this is better to understand now. We also explain the group composition now in more detail and the empathize phase won't be based on interviewing each other, as we initially planned.

P. 8-10 Line: 257-324
A didactic workshop concept will be developed based on the Design Thinking method mentioned above (see Figure 2).
As our participants may not be familiar with HL and ECAP, we will prepare materials to support the process of empathizing with the topics. Regarding ECAP, we will draw on the results of previous studies conducted within the HELICAP research group [40, 41] and consider the German Allergy Prevention Guideline as the most recent (2022) scientific source [9]. Regarding HL, the holistic model of Sørensen et al. [1] will serve as a basis in addition to the consensus study on HL practices and educational competencies for HPs [42]. To provide insight into the findings of the HELICAP group, we will draw on the materials developed during the first funding phase [43]. These include brochures, podcasts and factsheets. Participants will be invited to review these documents prior to their participation.

We will also share the results of our qualitative and quantitative study with the participants, providing further insight into the practices, barriers and facilitators of providing advice on ECAP in a HL-responsive way as well as the needs and wishes regarding further education in this area.

Organisation of the workshops:
After introducing the design thinking process, the research team will provide the participants with information about ECAP, HL and the results of our qualitative and quantitative study [15], thus enabling the group to define the focus, scope, and objectives of the intervention. Learning objectives for ECAP, HL and HL-responsive techniques will be jointly formulated. In addition, didactic methods will be provided to inspire the intervention, e.g. role play models, online learning tools, observational learning, inquiry-based learning, and game-based learning, etc.
Subsequently, the participants will collaboratively work in small groups of four to five people. Each group will consist of different participants, e.g. one student of midwifery, one practice instructor etc. (see table 1). In order to empathize with the specific needs of the group of midwives, e.g. students or practice instructors using different methods, e.g. interview and A-E-I-O-U-method (Empathize). This is the starting point for defining the needs that they will specifically address (Define). The groups will then start to develop ideas, without any restrictions regarding feasibility or economic considerations supported by applying the Walt Disney methods or 6-5-3-method. At the end of this phase, the groups will be asked to prioritize their favorite idea (Ideate).
In the next phase of the design thinking process, the participants will create a prototype of their favorite idea. They will be supplied with different materials (from story board wall to lego®) to creatively and hands-on put their ideas into reality and visualize them (Prototype). Key uncertainties of the proposed ideas for an intervention will be identified and the focus will now be on feasibility (see Figure 1). Lastly, each group will present their prototype to the other groups (Test). All participants are invited to provide feedback and ideas on how to enhance the presented prototype, i.e. ideas for intervention.

The workshops will be audio-recorded and transcribed. The research team conducting the workshop will closely follow the process and create an observation protocol. The focus will be on group interaction. The aim is to be able to track ideas that were either not considered or rejected during the development process and understand why these were rejected, e.g. due to group dynamics. These ideas may still be interesting and allow for more insight into specific needs.

10. Study Design – Line 288, Empathize is an important element of the Design Thinking. It should involve observe, engage, watch and listen. I don't think just talking to someone within the timeframe of a session in a workshop allows enough time to observe, engage, watch and listen to fully complete the 'Empathize' process.

Thank you for pointing this out. As mentioned above, we have already adapted our methods conducting the workshops. However, we would like to point out, that most of the participants are also at the same time the "target group", namely midwives. This means the empathizing process continues throughout the workshop and during every step. Therefore, we assume that the empathizing process can be realized within the timeframe of a workshop.

Additionally, we will also provide the participants with the results of our qualitative and quantitative study before the workshop, to offer further insights into practices,

barriers, facilitators of providing advice in a HL-responsive way, as well as needs and wishes regarding further education in this area.

P. 8 Line: 268-271: *We will also share the results of our qualitative and quantitative study with the participants, providing further insight into the practices, barriers and facilitators of providing advice on ECAP in a HL-responsive way as well as the needs and wishes regarding further education in this area.*

11. Study Design – Line 233 'protocols'. When you mentioned protocols as in 'document the process in detailed protocols, focusing on the ideas generated', it seems to refer to the process of your workshop. Here, the 'protocols' will then be edited… seems to refer to the protocol of the intervention. Please clarify. Also, does it mean that the ideate process will not only generate an idea but also the protocol?

As we have adapted our workshop design, this point is no longer applicable and we deleted this part.

12. Study Design – Line 234, 'completion'. What does it mean by 'completion'? Who will decide if an idea is 'complete' or not? I think the prototype is the next workshop?

As we have adapted our workshop design, this point is no longer applicable.

13. Study Design – Line 241 'discussed in plenary'. Does it mean there'll be small groups again?

As we adapted the workshop design, we hope this becomes clearer. Throughout the entire workshop the participants work in small groups. In the end, each group will present their prototype to the other groups. The prototype will jointly be discussed.

14. Study Design – How long will workshop 1 or 2 be? Is discussion of both workshops recorded and transcribed?

In the adapted workshop design, we plan to conduct workshops lasting approximately 7 hours (from 9am to 4pm). All the discussion (in the plenum, as well as in the small groups) will be audio recorded and transcribed. We included this in the manuscript.

P. 6-7 Line: 217-219: *We will conduct two co-design workshops with a duration of seven hours in two different locations (Mainz, Rhineland-Palatine and Leipzig, Saxony) in Germany […]*
P. 9 Line: 317: *The workshops will be audio-recorded and transcribed.*

15. Study Design – Workshop 2. The 'Prototype' and 'Test' of the Design Thinking method used in workshop 2 will have different meaning to health intervention. How do you adapt these steps to the health context?

The prototype is not intended to be the final intervention. The entire process of design thinking is meant to inform the intervention development in task 3. One of the prototypes may be (a part) of the final intervention, however we explicitly see this as a generation of ideas and not of the final intervention. In the phase "Test" we will discuss the ideas generated.

16. Study Design – Line 249-251. The core element 'programme theory' of the MRC framework is more than a logic model, it also identifies the mechanism: 'Programme theory describes how an intervention is expected to lead to its effects and under what conditions. It articulates the key components of the intervention and how they interact, the mechanisms of the intervention, the features of the context that are expected to influence those mechanisms, and how those mechanisms might influence the context.'

Thank you for pointing this out. We agree with you, that a logic model is only part of the program theory. We adapted the sentence accordingly:
P. 9 Line: 328-332: *In the next step, the research team will focus on the development of a program theory, to make explicit, how the intervention is intended to lead to its effects and under what conditions. A logic model focusing on resources / inputs, activities, outputs, and outcomes will be prepared to theoretically establish how the intervention is expected and intended to work [24, 40].*

17. Study Design – Line 253, 'qualitative study with midwives'. Are you referring to the study from your previous work?

Yes, we refer to the qualitative study we conducted in a previous study. We rewrote this to make it clearer and provided the reference.
P. 10 Line 334: *With the results of the previously conducted qualitative study with midwives [15]*

18. Study Design – Line 257, 'will be educational…' Would such an assumption affect your choice of intervention for development?

Thank you for this question. Yes, I think the assumption that the intervention will be educational, affected our choice of intervention development. I believe our mode of intervention development can lead to different formats, e.g. a toolkit, workshop or further education, but in the end, it will be our goal to educate midwives on how to provide advice on ECAP in a HL-responsive way, as we believe that this is how we achieve the most sustainable effects.

19. Study Design – Line 263, 'online workshop'. Would this be workshop 3? Given there are 3 workshops, how will you handle dropouts?

We didn't call it workshop 3 as it differs regarding aim and scope from the other workshops. This workshop is intended to be a short meeting, depending on the intervention developed, to present the intervention and ask for feedback and improvement. We will offer different timeslots to engage as many design thinking participants as possible to take part in this follow-up discussion. We believe design thinking workshop participants will be interested in the final intervention and thus assume that the dropout rate won't be too high.
We included the following sentence:
P. 10 Line 346-347: *Different timeslots will be offered to the design thinking workshop participants, to enable as many as possible to provide feedback on the final intervention.*

20. Study Design – Line 280, sample size of intervention. Is there any rationale for the sample size?

As it is still unclear, what the intervention will actually look like, it is not easy to estimate a sample size. However, we assume that the sample sizes we anticipate to pilot our intervention will provide insights into the process and outcome of the intervention. As we plan to conduct qualitative interviews, the sample size seems reasonable to be able to draw some conclusions about feasibility, effectivness etc. of the intervention. The sample size may be adapted, if it seems necessary according to the intervention developped.
We included this sentence:
P. 12 Line: 415-416: *Sample sizes may be adapted if needed.*

21. Study Design – Line 280, what is the difference between participants a (in practice) and participants b (as practice guide)?

In Germany it is part of the professional education of future midwives to accompany experienced and especially trained midwives during their works (e.g. antenatal or postnatal visits within the home of families). Those midwives are called practice instructors (or practice guides as was our first translation), as they teach future midwives within their working environment. These midwives have an additional education. We included a more detailed explanation when describing the sample.
P. 7 footnote 1: A midwife who has obtained an additional pedagogical qualification that enables her to provide structured guidance, supervision, and assessment of midwifery students during their clinical training. This role requires both professional experience and formal training in practice instruction.

22. Study Design – Line 289, about delivery of intervention. As the participant deliver the intervention, will someone be at the receiving end of the intervention? How will you recruit people who's on the receiving end? Will these people on the receiving end also be interviewed?

This seems to be formulated in a misleading way. It is our intention to develop an intervention that is educational but does not solely depend on us (the research team) to be delivered. Thus, we aim at providing materials that enables others to conduct this educational intervention. This could be a module that could be integrated in midwifery studies, or a workshop. We plan to develop materials that can be used by anyone providing education for midwives. In order to test these materials we plan to have someone, use them and provide feedback if this is feasible and what could be adapted, however if this proves difficult, it will be one of the research team to conduct the intervention in the beginning.

We rewrote this section to make this clearer:
P. 11 Line: 373-381:
In order to gain insight into the process of the conduct intervention, either one of the research team will conduct the intervention or be present and closely observe how the intervention is conducted by a third person, e.g. from an educational institution using observation protocols. Semi-structured interviews will be undertaken with people involved in conducting of the intervention in order to gain insight into their experiences. The focus will be on barriers and facilitators, as well as the feasibility of the intervention.
Furthermore, the intervention will be evaluated focusing on the perspective of the participants receiving the intervention [47].

23. Study Design – Line 290, interviews. How many interviews are planned?

We assume a minimum of three interviews per group (midwives, midwives working as practice instructors, students of midwifery) will be conducted. We included this in the manuscript.
P. 11 Line: 382-384: *The aim is to conduct interviews with at least three participants receiving the intervention of each group (midwives, midwives working as practice instructors, midwifery students).*

24. Study Design – Line 294, 'member-checking'. What do you mean by 'member-checking'? Do you mean people receiving the intervention?

Yes, this is what we meant. We understand this is unnecessary and rather misleading. Therefore, we deleted member-checking.

25. Study Design – Line 296, 'interviews'. Which interview? I think you have proposed 2 interviews, one with the person delivering the intervention and another which I am not quite sure who you referred to.

We plan to conduct interviews with the people delivering the intervention, e.g. teaching staff at universities and we plan to conduct interviews with persons receiving interviews, e.g. students of midwifery science. We rewrote this section and hope this is clearer now.
P. 11 Line 373-394

26. Study Design – Lines 304-305 interview data. How many researchers will be involved in the analysis of the interview data?

Two researchers, supported by a student assistant, will be involved in analyzing the interview data.
We have included this information as follows:
P. 11 Line 392-393: *All interviews will be audio-recorded, transcribed, and subjected to content analysis performed by two researcher and a student assistant.*

27. Study Design – Line 310, quantitative data. The sample size is small, has your sample size calculation ensure that you have enough power to detect changes?

We will try to recruit as many participants as possible. However, we are only piloting the intervention and we will emphasize this fact when publishing our results. To evaluate the anticipated intervention, we clearly need a larger sample size than is possible in the piloting planned within this study.

28. Study Design – Line 315, simulation lab. What is the purpose of this additional testing? The description isn't too clear about the procedure. What does it mean that only one group will participate in the educational intervention? Do you mean there'll be an intervention group and a control group? How many participants are you expecting?

Thank you for pointing out that this wasn't described in an understandable way. Yes, we would like to work with two groups of students, as in intervention and control groups. We would like to understand the changes regarding knowledge and competences (P. 12, line 410-413) that occur when students are educated with our intervention compared to students that are educated in the traditional way. We added this to our description:

P. 12 Line: 408-410*: Prior to this, only one of the groups will participate in the educational intervention, the control group will be educated in the traditional way.*

29. Study Design – Do you think your evaluation needs to be adjusted when you have decided on the intervention?

This can very well happen, and we are open to adjust our evaluation depending on the intervention that is being develop. The design thinking workshops are central to our study. It is not just the prototype being developed, but all steps, to better understand needs and wishes, potential barriers and facilitators in supporting midwives to provide advice to all families in a health literacy responsive way on ECAP, but also other health related topics. We are curious what kind of intervention will be developed in the end and we believe iterative processes adapting and reconsidering a central to receive a good result.

To make this clear in the manuscript as well, we included the following sentence:
P. 10 Line 358-359: *Changes in our piloting and evaluation plan may occur depending on the intervention that will be developed.*

30. Study Design – Line 323, the sentence is incomplete.

Thank you for pointing this out. We added the word: adapted.

31. Patient and Public Involvement Statement – I think you can also mention the workshop participants (the midwives) as public involvement.

Thank you for your opinion on this. We were struggling with this, as midwives can be seen as experts, but we agree with you as they are the target group, they can also be seen as public involvement. We included this in the statement (P. 12, Line: 419-423).

Minor comments:
1. Target group vs target groups – Throughout the manuscript, you have 'target group' and you have 'target groups'. I think you have midwives as your target group, do you also have other target groups? It is confusing.

Thank you for pointing this out. As we rewrote multiple sections and changed the wording, we hope this also becomes clearer now. We have midwives, midwives working as practice instructors and midwifery students as target groups. All of them are midwives in some respect, however these groups differ significantly. This is why we use the expression target groups.

2. Background – First paragraph, line 66, the term 'use case', I am not sure what you mean by 'use case'.

Thank you for pointing out, that this is not understandable. We rewrote this sentence and hope this makes it clearer:
P. 2 Line: 73: *Since 2020, the HELICAP (Health Literacy in Early Childhood Allergy Prevention) research group focuses on HL with early childhood allergy prevention (ECAP) as primary example.*

3. Background – First paragraph, lines 66-69, the sentence is too long and difficult to read.

Thank you for pointing this out. We shortened the sentence and hope it is better to read now:

P. 2 Line: 73-76: *HL is understood as the knowledge, motivation and competence to access, understand, appraise and apply health information. However, this is not limited to the skills of the individual, but perceived in a broader context (cf. Figure 1) including situational, societal and environmental determinants.*

4. Background – Second paragraph, line 74, please check how numbers are reported as per journal's guidelines.

We mistakenly used the German way of reporting numbers. We changed it from 1.000 to 1,000. We changed this also on P. 6, Line 200: 27,000 midwives instead of 27.000,

5. Background – Lines 74-75, the sentence 'Research indicates…', the way this sentence is written makes me think that it is the newborn who should adopt certain health behaviours. I think you mean the mother needs to adopt certain health behaviours, is that correct? Please rewrite to clarify.

Thank you for pointing this out. We rewrote the sentence as follows:
P. 2 Line 81-82: Research shows that families can lower their children's risk of developing allergies by adopting certain health behaviours during the first 1,000 days of life.

6. Study Design – line 112, 'cf. Figure 1', I'm not sure why you use 'cf.'. To ask readers to see a figure, you can simply say 'see Figure 1'. Besides, Figure 1 is your study process, not just problem identification, so, you need to make it clear like '(Problem Identification, see Task 1 of Figure 1)'.

Thank you for this suggestion. We changed it accordingly!

7. Study Design – Lines 134-135, the term 'representative insight' is not a term commonly used in English even though I know what you mean.

We rewrote the sentence and had it checked by a native speaker. We hope it is better to understand now:

P. 5 Line: 154-158: We will conduct an exploratory quantitative study, using an online survey to obtain representative data on how midwives provide HL-responsive advice on ECAP by midwives. This will provide insight into the context and a starting point for the development of the intervention, focusing on the specific needs of the target group midwives (Problem Identification, see Task 1 of Figure 1)

8. Study Design – the '70-items questionnaire' should be '70-item questionnaire'.

We changed this as suggested.

9. Study Design – Line 164 the subheading of Task 2, I'd suggest using the same wording as in Figure 1.

We changed this as suggested.

10. Study Design – Lines 216 and 236, description of the workshops, I'd suggest just use 'Conducting of co-design workshop 1 (Idea generation), then, 'Conducting of co-design workshop 2 (concretization). Try not to create too many different terms for your co-design workshops.

As we changed the entire workshop sessions, this is not applicable anymore.

11. Study Design – Line 282, 'conduction'. I don't think this is the right word. Do you mean 'implementation'? You have used 'conduct' in other parts of the manuscript as well.

We changed this as suggested.

Reviewer: 1
If you have selected 'Yes' above, please provide details of any competing interests.: Not applicable

---

VERSION 2 - REVIEW

**Reviewer**	**1**
**Name**	**Cheng, Christina**
**Affiliation**	**La Trobe University**
**Date**	**11-Aug-2025**
**COI**	

Thank you for revising the manuscript and clarifying issues previously identified in the manuscript. I see that you have changed HL-sensitive to HL-responsive, which I believe is much better as it is more commonly used in English. However, despite your efforts to edit the manuscript, I still find the manuscript not easy to read and there are a lot of guessing in trying to understand what you actually mean. I would highly recommend you using the language editing services available at BMJ Open. You have made some of your Methods clearer now, however, I still have questions about your Methods.

Major comments:

1. Strengths and Limitations of the study – The simulation laboratory is still a method that you used, not a 'strength'. What you need to present here is why simulation laboratory is a strength? More comment about 'simulation laboratory' below.

2. Study design – Sorry that I missed this issue last time. I think you should provide a definition of health literacy-responsive advice. That will provide a clearer context for your study.

3. Study design – Task 1, Given it is a 70-item questionnaire, I recommend you talking to a psychometrician to conduct a validity testing of the questionnaire to determine its validity and reliability. It is important to demonstrate that you are using a questionnaire that actually measures the data that you want, demonstrating content validity is not enough.

4. Study design – Task 4, Outcome evaluation. Following on my comment above, you mentioned that you'll be using a shorter version of the questionnaire. In such a case, it is even more important to conduct a validity testing of the questionnaire and this shorter version as well. You need validity evidence to demonstrate that your questionnaire is measuring what you intend to measure as well as it is a questionnaire that is sensitive enough to detect changes.

5. Study design – Task 4, Outcome evaluation. The simulation laboratory just comes out of the blue. You have not indicated that there will be an intervention group and control group until the second paragraph of the Outcome evaluation, even though you briefly mentioned about quasi experiment in the Strengths and Limitations. Your sample and recruitment section also does not indicate that you have plans to have 2 groups. You have also not provided the rationale for using a simulation lab. Why do you think a simulation lab is a better way to deliver your intervention? Or do you mean there will be 2 stages of the intervention. One is to conduct the intervention in the conventional way, then, the second stage is to have the intervention group using the simulation lab and the control group using the conventional way? A lot of details about doing a quasi experiment are simply missing in your Outcome evaluation.

Minor comments:

6. Abstract – Ethics is missing.

7. Title – It is better to spell out MRC instead of using an acronym as people may not know what MRC means.

8. Study design – Task 1, last sentence of first paragraph 'The 70-item questionnaire…' With this sentence, I expect a reference of this previous study at the end, but your next 2 paragraphs went on to describe the development of this questionnaire. So, consider moving it further down.

9. Study design – Task 1, line 161, I am not sure what you mean by 'wishes'.

10. Study design – Task 1, line 181-182, the meaning of this sentence 'Response options were mostly used in full' is not clear.

VERSION 2 - AUTHOR RESPONSE

Reviewer: 1
Dr. Christina Cheng, La Trobe University

Comments to the Author:
Thank you for revising the manuscript and clarifying issues previously identified in the manuscript. I see that you have changed HL-sensitive to HL-responsive, which I believe is much better as it is more commonly used in English. However, despite your efforts to edit the manuscript, I still find the manuscript not easy to read and there are a lot of guessing in trying to understand what you actually mean. I would highly recommend you using the language editing services available at BMJ Open. You have made some of your Methods clearer now, however, I still have questions about your Methods.

Dear Christina Cheng,
Thank you for reviewing our manuscript again and thank you for your further comments. Please see our responses to your comments below. Our changes are marked in the manuscript.
To improve the language and readability we have commissioned a translator who has worked for us before to proofread the manuscript. We hope it is now easier to read.

Major comments:
1. Strengths and Limitations of the study – The simulation laboratory is still a method that you used, not a 'strength'. What you need to present here is why simulation laboratory is a strength? More comment about 'simulation laboratory' below.

Thank you for pointing this out. We deleted the simulation laboratory as a strength. We rewrote the section and it reads as follows:
Page 2, line 62-64: *Testing the intervention in different target groups provides early insights into feasibility and preliminary outcomes, which can help us refine the intervention before conducting a larger trial.*

2. Study design – Sorry that I missed this issue last time. I think you should provide a definition of health literacy-responsive advice. That will provide a clearer context for your study.

Thank you for pointing this out. We agree that defining health literacy-responsive advice would improve understanding of the context of our study. We have now included a definition:
Page 3, Line 102-105*: In our understanding, HL-responsive advice comprises the communication of evidence-based health information in a way that enables people to understand, appraise, and apply this information with a view to engaging and supporting them in making health-related decisions.*

3. Study design – Task 1, Given it is a 70-item questionnaire, I recommend you talking to a psychometrician to conduct a validity testing of the questionnaire to determine its validity and reliability. It is important to demonstrate that you are using a questionnaire that actually measures the data that you want, demonstrating content validity is not enough.

Thank you for pointing this out. Ideally, our manuscript on the questionnaire design would have been published by now, to provide a detailed overview of the design of the questionnaire and the piloting. Unfortunately, this is not the case. Items were construed focusing on midwives' knowledge, skills, attitudes and practices, in order to quantify in detail the aspects considered relevant when providing advice on ECAP,

in howfar HL is considered, and what is needed to support midwives in providing HL-sensitive ECAP advice.
We ensured that content validity was given, that distributions were appropriate and that items measuring a construct had an acceptable Cronbach's alpha. Further psychometric testing was without the scope of our research. We have now included further information in the manuscript (see below).
Additionally, we need to mention, that data collection on a larger scale using this questionnaire took place from November 2024 to January 2025. We had to begin our study as we only have three years of funding and could not wait for the publication of the study protocol and the reviewer comments.

Page 5 Line 178-191: *We calculated the mean and standard deviation and examined the response distributions for floor/ceiling effects and response bias patterns. For most items, the response options were fully used and distribution was acceptable. Two items showed a ceiling effect (topics considered important during the provision of advice on ECAP): With regard to ECAP, breast-feeding was considered important by almost all midwives, as was smoking, however, we decided to keep these items in the questionnaire to provide a complete picture of topics. Two items focusing on how advice on ECAP is provided during consultations were designed to be mutually exclusive, however cross-tabulation showed that this was not the case. These items were therefore rephrased. To check internal consistency, we calculated Cronbach's alpha for the following constructs: importance of topics in consultations on ECAP (11 items, five-point-Likert scale, $\alpha = 0.81$), professional HL regarding ECAP (four items, five-point-Likert scale, $\alpha = 0.78$), HL-responsive advice (seven items, five-point-Likert scale, $\alpha = 0.77$), perception of midwifery work in regard to providing advice on ECAP in an HL-sensitive way (eight items, five-point-Likert scale, $\alpha = 0.76$).*

4. Study design – Task 4, Outcome evaluation. Following on my comment above, you mentioned that you'll be using a shorter version of the questionnaire. In such a case, it is even more important to conduct a validity testing of the questionnaire and this shorter version as well. You need validity evidence to demonstrate that your questionnaire is measuring what you intend to measure as well as it is a questionnaire that is sensitive enough to detect changes.

Thank you for pointing this out. We have now provided a more explicit explanation of our validity testing of the questionnaire (see above). The questionnaire will be adapted once the focus of the intervention is determined and the intervention development is completed, to ensure changes can be measured by using this instrument. Additionally, we want to point out, that the questionnaire is only one part of our evaluation among qualitative interviews, process evaluation and the observation in simulation laboratories.

Page 10 Line 374-377: *To evaluate the outcome of the intervention, we will develop a short version of the questionnaire on providing HL-responsive advice. We will do this once the focus of the intervention has been established and its development completed to ensure the changes brought about by the intervention can be measured.*

5. Study design – Task 4, Outcome evaluation. The simulation laboratory just comes out of the blue. You have not indicated that there will be an intervention group and control group until the second paragraph of the Outcome evaluation, even though you briefly mentioned about quasi experiment in the Strengths and Limitations. Your sample and recruitment section also does not indicate that you have plans to

have 2 groups. You have also not provided the rationale for using a simulation lab. Why do you think a simulation lab is a better way to deliver your intervention? Or do you mean there will be 2 stages of the intervention. One is to conduct the intervention in the conventional way, then, the second stage is to have the intervention group using the simulation lab and the control group using the conventional way? A lot of details about doing a quasi experiment are simply missing in your Outcome evaluation.

Thank you for this very important remark. It demonstrates that we need to explain the training of midwives in Germany more thoroughly and introduce this earlier in the manuscript, in order to make clear, how we plan to use the simulation lab in our evaluation of the intervention.

We have included the following in the introduction, to provide a clearer picture of midwifery education in.

Page 3-4, Line 113-123: *Since 2020, midwifery in Germany requires a university education and is now offered as a dual Bachelor's degree, combining classroom instruction and a practical placement. Germany therefore currently has midwives who were trained in vocational schools (until 2020), midwives who were trained in vocational schools and are now working as practice supervisors for future midwives studying midwifery as well as midwifery students who are in the process of completing a university degree. The academic education of midwives now combines at least 2,200 hours of theoretical instruction with 2,200 hours of clinical practice. Clinical placements are undertaken in collaboration with hospitals and community-based midwifery practices, under structured clinical supervision. In addition, simulation laboratories are a compulsory part of the midwifery curriculum, serving as a bridge between classroom teaching and clinical placements by providing a safe environment in which to practice and demonstrate counselling skills.*

We rewrote the section on outcome evaluation to make clarify how we envisage using the simulation laboratory, as we do not currently plan to deliver the intervention there, but rather to test it.

Page 10, Line 383-399: *In addition, within the subgroup of midwifery students at the University of Mainz, we will assess the intervention's effects on knowledge and competence regarding the provision of advice on ECAP in an HL-responsive way. Therefore, half of the participating students will receive their training based on the intervention developed and the other half of the students will receive their training according to the existing curriculum. Subsequently, all students will engage in simulated consultation sessions on ECAP. The students will be given the task of providing advice on ECAP to a family (played by actors) in the simulation laboratories. The latter provide a controlled environment in which to practice consultations in realistic but standardized scenarios. The knowledge and proficiency levels of the members of both groups will be evaluated according to a predefined checklist (objective structured clinical examination, OSCE). This checklist includes the horizons of expectations regarding ECAP in a consultation situation, as well as the corresponding grading schemes that are commonly used. The controlled and standardized environment of the simulation laboratory will enable us to gain a thorough understanding of the differences in knowledge and consultation skills between students who have received the intervention and those who have not. Based on the results of the process and outcome evaluations, the intervention will be adapted if necessary.*

Minor comments:
6. Abstract – Ethics is missing.

We included the ethics now in the abstract as well on page 2, line 48-51.

7. Title – It is better to spell out MRC instead of using an acronym as people may not know what MRC means.

Thank you, we changed the titel accordingly.

8. Study design – Task 1, last sentence of first paragraph 'The 70-item questionnaire…' With this sentence, I expect a reference of this previous study at the end, but your next 2 paragraphs went on to describe the development of this questionnaire. So, consider moving it further down.

Thank you! As we cannot provide a reference (yet), as the manuscript is still under review, we moved the sentence further down as you suggested.

9. Study design – Task 1, line 161, I am not sure what you mean by 'wishes'.

Thank you, we changed this following the suggestions of our translator:
Page 4, line 151-153: *We will also collect data on the needs, wishes of the respondents when it comes to providing advice, as well as certain sociodemographic characteristics of the respondents.*

10. Study design – Task 1, line 181-182, the meaning of this sentence 'Response options were mostly used in full' is not clear.

Thank you, we changed this following the suggestions of our translator:
Page 5, line 179-180: *For most items, the response options were fully used and the districution was acceptable.*

VERSION 3 - REVIEW

**Reviewer**	**1**
**Name**	**Cheng, Christina**
**Affiliation**	**La Trobe University**
**Date**	**25-Oct-2025**
**COI**	

Thank you for revising the manuscript and addressing my comments. You have adequately addressed my concerns. Just need to do some more editing and proofreading as there are still some minor language issues. For example, there are two 'with's in the last sentence of the Introduction of the abstract. Or, the second sentence of the Introduction in the main text, 'Since 2020, the Health Literacy in Early Childhood Allergy Prevention (HELICAP)

research group has been focusing on HL using early childhood allergy prevention (ECAP) as the main use case', it is better written as 'Since 2020, HL using early childhood allergy prevention (ECAP) has been the focus of the Health Literacy in Early Childhood Allergy Prevention (HELICAP) research group.' Also, I can see there are still some 'HL-sensitive' which has not been revised to 'HL-responsive'. Otherwise, good luck with your manuscript and study.

VERSION 3 - AUTHOR RESPONSE

Reviewer: 1
Dr. Christina Cheng, La Trobe University
Comments to the Author:
Thank you for revising the manuscript and addressing my comments. You have adequately addressed my concerns. Just need to do some more editing and proofreading as there are still some minor language issues. For example, there are two 'with's in the last sentence of the Introduction of the abstract. Or, the second sentence of the Introduction in the main text, 'Since 2020, the Health Literacy in Early Childhood Allergy Prevention (HELICAP) research group has been focusing on HL using early childhood allergy prevention (ECAP) as the main use case', it is better written as 'Since 2020, HL using early childhood allergy prevention (ECAP) has been the focus of the Health Literacy in Early Childhood Allergy Prevention (HELICAP) research group.' Also, I can see there are still some 'HL-sensitive' which has not been revised to 'HL-responsive'. Otherwise, good luck with your manuscript and study.

Dear Christina Cheng,
Thank you for reviewing our manuscript again. We deleted "with", changed the sentence according to your suggestion and replaced "HL-sensitive" by "HL-responsive".
We wanted to thank you again for your thorough and detailed review of our study protocol. We believe it is highly improved now.
